# Influenza A Viral Infection with Septic Shock in Pregnancy

**DOI:** 10.1155/2019/2470352

**Published:** 2019-04-21

**Authors:** Soe Lwin, Myat San Yi, May Shi Leong, Haris Suharjono, Tin Moe Nwe

**Affiliations:** ^1^Department of Obstetrics & Gynecology, Faculty of Medicine and Health Sciences, UNIMAS, Malaysia; ^2^Department of Obstetrics & Gynecology, Sarawak General Hospital, Malaysia; ^3^Department of Basic Health Sciences, Faculty of Medicine and Health Sciences, UNIMAS, Malaysia

## Abstract

The influenza virus is RNA virus and is classified into four subtypes, influenza A, influenza B, influenza C, and influenza D. One of the subtypes of influenza A, the H1N1 strain, also known as swine flu, is especially of high risk for development of complications in pregnant women. The influenza A virus infection is difficult to diagnose clinically because its presenting symptoms are similar to those of the common cold but are more severe, last longer, and can be potentially life-threatening. This case also presented with common cold symptoms but her condition worsened later. Fortunately, obstetric health providers were vigilant enough to address the developing infection and its related complications. It was the cooperative effort of multidisciplinary team care which resulted in a favourable outcome in both mother and baby.

## 1. Introduction

Influenza virus is a single-stranded, enveloped RNA virus from* Orthomyxoviridae* family. Its incubation period is 2-5 days and the transmission is mainly air-borne, i.e., droplet inhalation or direct transmission or contact through hands and fomites. It is classified into four distinct generations such as influenza A, B, C, and D depending on antibody responses to glycoproteins,* hemagglutinin* (HA), and* neuraminidase* (NA) on the surface of the viruses [1].

One of the subtypes of influenza A, influenza A (H1N1) strain called swine flu was first identified in April 2009 and the outbreak has since reached pandemic status at that time [2, 3]. So far, there have been 4 pandemic attacks worldwide: Spain in 1918 (unknown strain but suggestive of avian-like H1N1), Asian flu in 1957 by H_2_ N_2_, in Hong Kong in 1968, and Mexico in 2009. After each pandemic attack, healthcare personnel have become more aware of its lethal complications. In Malaysia, the avian influenza virus (AIV) (H5N1) outbreaks occurred in 2004, 2006, 2007, and 2017 in the state of Kelantan, Perak, and Pulau Pinang [4].

The influenza virus infection presents with fever, cough, sore throat, rhinorrhea, headache, myalgia, vomiting, and diarrhea. Pregnant women are at especially high risk of the development of complications of H1N1 influenza A. This increased risk is related to several physiological changes during pregnancy including alterations in the cardiovascular, respiratory, and immune systems. The serious illness and hospitalization rates of women with influenza during pregnancy have a 4- to 5-fold increase compared to nonpregnant women [2, 5–8]. Qi et al. (2014) stated that pregnant women are particularly susceptible to severe complications from influenza and have a greater mortality risk.

Influenza A infection is diagnosed by obtaining an upper respiratory specimen (nasopharyngeal swab, nasal aspirate, or a combined nasopharyngeal swab with oropharyngeal swab or throat swab) to test for novel influenza A (H1N1) virus. The specimen should be placed into sterile viral transport media and immediately placed on ice or cold packs or at 4°C (refrigerator) for transport to the laboratory and the virus is identified by real-time polymerase chain reaction (RT-PCR) or viral culture method [9, 10].

## 2. Case Presentation

A 26-year-old female, period of gestation of 35 weeks and 6 days into her third pregnancy, presented with contraction pain which was increased in intensity and frequency. She denied any leaking liquor nor show. Fetal movements were well felt. She claimed that she had fever, headache, sore throat, and dry cough for one-day duration. She denied any travelling or any contact with live poultry. She also had no history of dysuria and frequency. Her booking visit was at 9^th^ week of gestation and all routine antenatal tests were negative. Latest scan was done at 33-week follow-up and all parameters were corresponding to gestational age.

On physical examination, she appeared alert, conscious, with no tachypnea and no signs of dehydration. She was afebrile, with no cervical lymph nodes and tonsillar enlargement. The lungs were clear. She had tachycardia up to 110/min and blood pressure was 105/57 mmHg. The abdominal findings corresponded to gestational age, single fetus, longitudinal lie, and cephalic presentation with good fetal heart rate. The estimated fetal weight was 2 to 2.2 kg. The vaginal examination noted that os was closed. The ultrasound examination was repeated and it corresponded to respective gestation. The urine dip stick was suggestive of urinary tract infection. The urine specimen was sent for culture and sensitivity (C&S) before starting the antibiotics.

She was observed in the maternity ward. However, the patient was noted to be febrile with temperature of 39°C, tachycardia, and low blood pressure 80/50mmHg on next day leading to septic shock. Her blood pressure was maintained by intravenous noradrenaline infusion. A septic workup was done and initial resuscitative measures were performed. The blood test results came back with white cell count of 15.34 x 10^9^/L and C-reactive protein level of 365mg/L. She went into active phase of labour at the same time.

**Figure 1:**
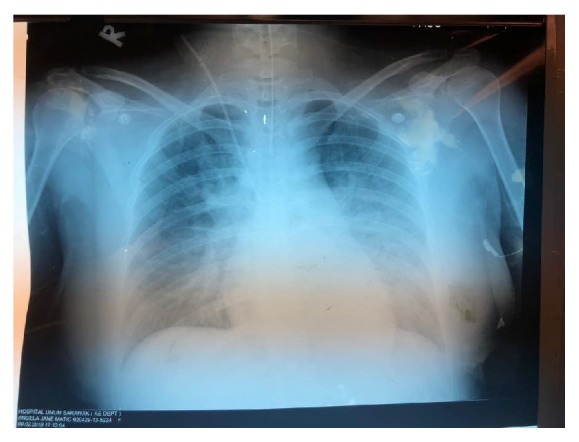


**Figure 2:**
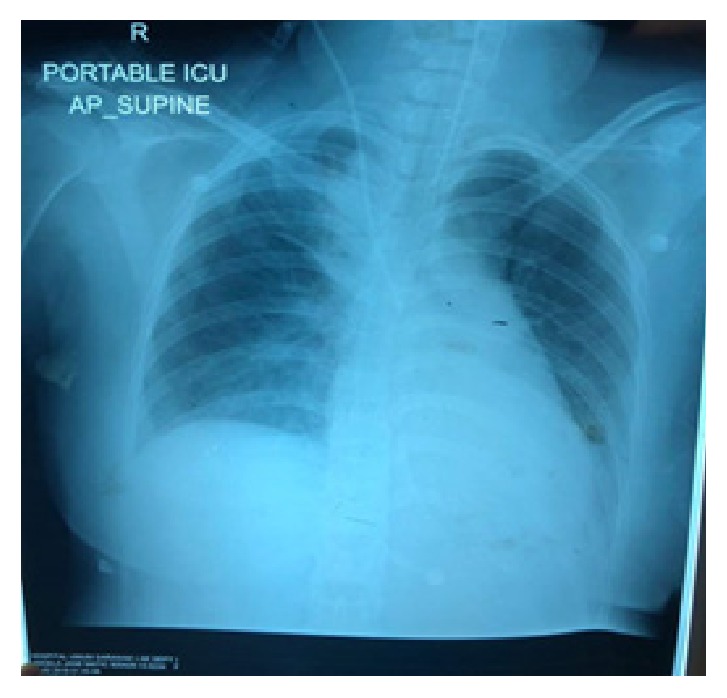


Multidisciplinary input from anaesthetist for ICU backup, physician, and infectious disease teams was obtained, led by obstetric consultant shared care. The arterial blood gas showed metabolic acidosis. Her condition worsened as she became restless and breathless even with 2L/min oxygen under nasal prong. Four hours in labour, it was noted that the progress of labour was unsatisfactory with features of fetal distress. A decision was made to deliver by caesarean section in collaboration with anaesthetist and ICU team. An alive, female, 2.61 kg baby with Apgar score of 5 in 1 minute, 7 in 5 minutes and 8 in 10 minutes, was delivered and estimated blood loss was about 700 ml. A placental swab was taken for C & S. The anaesthetist noted on lungs auscultation that air entry at the left lower zone and a portable chest X- ray was ordered. The chest X-ray was reported as slight basal lobe collapse at left lobe with reactive pleural effusion (Figures 1 and 2). Tamiflu was started and she was diagnosed with Acute Respiratory Distress Syndrome (ARDS). The baby was admitted to the nursery for late prematurity with presumed sepsis. Antibiotic treatment was given. The placenta swab culture result came back as sterile; still the baby needed photo therapy as neonatal jaundice arose.

The patient was extubated after one day but continued support with Venturi mask 50% and 1.5L/min of oxygen. Once stable, she was transferred to High Dependency Unit and continued monitoring there. The throat swab was reported as influenza A positive and the message was relayed to both Infectious Diseases and Paediatric team. The recommendation was to continue tablet Tamiflu (oseltamivir phosphate) for one week. Universal precaution and isolation of the patient were followed. The symptoms subsided with the treatment. The patient and baby were discharged on the fifth day postoperatively without complications.

## 3. Discussion

The patient presented with a seasonal flu; however, her conditions deteriorated quickly into septic shock with ARDS. Despite this, early awareness of the serious symptoms and a multidisciplinary team approach with appropriate and aggressive treatment resulted in favourable outcome for both mother and baby. Unfortunately, the subtyping of influenza A was not able to be carried out as our center lacked such facility. However, it did not change the management and its good outcome.

There are warning signs which should alert the clinicians to the diagnosis of an influenza A infection. Early recognition of warning signs will lead to timely treatment and a better prognosis.

Warning signs for the clinician are as follows:

- Difficulty breathing or shortness of breath
- Pain or pressure in the chest or abdomen
- Sudden dizziness
- Confusion
- Severe or persistent vomiting
- Flu-like symptoms that improve but then return with fever and worsening cough

(From flu symptoms: https://www.cdc.gov/flu/takingcare.html [11].)

## 4. Antiviral Medication

Pregnancy is a physiological state of relative immunosuppression and all pregnant women should be assumed to be at high risk and should be treated if the potential benefit outweighs the theoretical risk of the fetus. Antiviral drugs will help to relieve symptoms and reduce viral shedding, but therapy is more beneficial if started within 12-48 hours. Neuraminidase inhibitors are used and they are as an adjunct to immunization. Oseltamivir is taken orally and zanamivir is administered via inhalation. Both are category C drugs but oseltamivir is preferred over zanamivir due to its well-evidenced results from clinical experience with a guaranteed systemic absorption [9, 10]. See Table 1.

## 5. Isolation

The patients should continue to practice good hand hygiene and cough etiquette and wear a face mask for the next 7 days. The patients with suspected N1H1 should wear a face mask and be placed in an isolated room away from providers and other hospitalized patients [9]. Surveillance on the appearance of new symptoms as well as progress of current condition is necessary during isolation period.

## 6. Breastfeeding

Breastfeeding is encouraged in H1N1 influenza although H1N1 transmission through breast milk is unknown; breastfeeding strengthens the neonatal immune response and infants who are bottle fed may be prone to getting a viral infection [13]. If the infant needs to be isolated from the infected mother, the infant should receive bottle feedings of expressed breast milk until the mother and infant can be reunited. The patients should use a facemask and practice strict hand hygiene and cough etiquette [9, 10].

## 7. Immunization

Immunization is recommended to pregnant women especially during flu season (November–March). They are low-cost interventions that have been shown to have substantial benefits for both mother and baby [14, 15].

## 8. Life Style Changes

Personal hygiene is important in preventing transmission of viral influenza. Measures such as hand hygiene and wearing a face mask or N95 disposable respirator during close contact with a sick individual should be advised to patients and relatives. Household members should be monitored for influenza-like symptoms and should be advised to contact their healthcare provider if symptoms occur [16].

## 9. Conclusion

Pregnant women are at high risk of infection with the H1N1 influenza A virus at high risk of spontaneous miscarriage and associated neural tube defects, neonatal seizures, encephalopathy, cerebral palsy, and neonatal death [17–19]. The influenza A virus infection is difficult to be diagnosed clinically because the presenting symptoms of an influenza infection are similar to those of the common cold but they are more severe and protracted and can potentially be life-threatening. Therefore, early recognition and awareness of warning signs with appropriate, timely treatment, and multidisciplinary support matter to achieve the best outcome.

## Figures and Tables

**Table 1:** 

Drug	Treatment	Chemoprophylaxis

Oseltamivir	75 mg twice a day for 5 days	75 mg once a day for 10 days
Zanamivir	5 mg inhalations twice per day for 5 days	5 mg inhalations daily for 10 days
Peramivir	One time intravenously over 15-30 mins	

From http://www.cdc.gov/h1n1flu/recommendation/html [12].

## Abbreviations

AIV: Avian influenza virus
ARDS: Adult respiratory distress syndrome
CDC: Centers for Disease Control
C& S: Culture and sensitivity
CTG: Cardiotocogram
ICU: Intensive care unit
ID: Infectious disease
LSCS: Lower segment caesarean section
O&G: Obstetrics and gynecology
RT-PCR: Real-time polymerase chain reaction
US: United States
WHO: World Health Organization.
